# From Childhood to Adulthood: A Case of Congenital Tracheal Stenosis

**DOI:** 10.7759/cureus.97763

**Published:** 2025-11-25

**Authors:** Inês Duarte, Filipa Canedo, Mario Pinto, Nicole Murinello, Antonio Miguel

**Affiliations:** 1 Pulmonology, Hospital Santa Marta, Unidade Local de Saúde São José, Lisbon, PRT

**Keywords:** adult follow-up, congenital tracheal stenosis, conservative management, pulmonary artery sling, rare

## Abstract

Congenital tracheal stenosis (CTS) is a rare and potentially life-threatening malformation, often associated with cardiovascular anomalies, such as pulmonary artery sling. We report a 20-year-old woman with a history of pulmonary artery sling surgically corrected in infancy, who presented with diffuse CTS. Early symptoms of dyspnea, wheezing, and episodes of airway obstruction prompted evaluation, and imaging with computerized tomography and flexible bronchoscopy confirmed complete tracheal rings with a lumen of 6-8 mm. Pulmonary function tests showed fixed airway obstruction and flattening of inspiratory and expiratory flow-volume curves. At 19 years, she remained symptomatic with exertional stridor but preserved exercise tolerance, and follow-up imaging demonstrated stable diffuse stenosis. Given relative clinical stability and diffuse involvement unsuitable for endoscopic intervention, conservative management with regular multidisciplinary follow-up and symptomatic therapy was pursued. This case highlights the diagnostic and therapeutic challenges of CTS in adulthood and emphasizes the importance of early recognition, functional assessment, and individualized multidisciplinary management. Further studies are needed to determine long-term outcomes and optimal surveillance strategies in adults with persistent diffuse tracheal stenosis.

## Introduction

Congenital tracheal stenosis (CTS) is a rare malformation, with an estimated incidence ranging from 0.2 to 2 per 100,000 live births. It can be life-threatening, as it is characterized by a fixed narrowing of the tracheal lumen. This obstruction results from the presence of complete tracheal cartilaginous rings rather than the normal C-shaped configuration [1-5]. CTS is commonly associated with cardiovascular anomalies; among these, pulmonary artery sling is particularly important, given its frequent occurrence in children with CTS and its significant contribution to airway compromise [5]. This condition is most frequently diagnosed in neonates or early childhood, when limited airway reserve leads to early presentation with respiratory distress, stridor, or recurrent infections. In contrast, adults may remain asymptomatic or experience only mild, nonspecific respiratory symptoms, reflecting compensatory airway growth and adaptation over time [1,6]. The clinical spectrum is broad and largely determined by the severity, location, and extent of the stenosis, ranging from silent disease to significant airway compromise [3]. In milder forms, patients may remain stable into adulthood without major complications [6]. We report a case of CTS in adulthood.

## Case presentation

The 20-year-old patient was diagnosed with a congenital cardiovascular malformation and pulmonary artery sling in infancy and underwent surgical correction at three months of age. Soon after, she began to experience episodes of choking, which raised suspicion of a worsening associated airway abnormality. At five years of age, she was followed in a pediatric pulmonology clinic, where a chest X-ray showed narrowing of the tracheal lumen.

**Figure 1:**
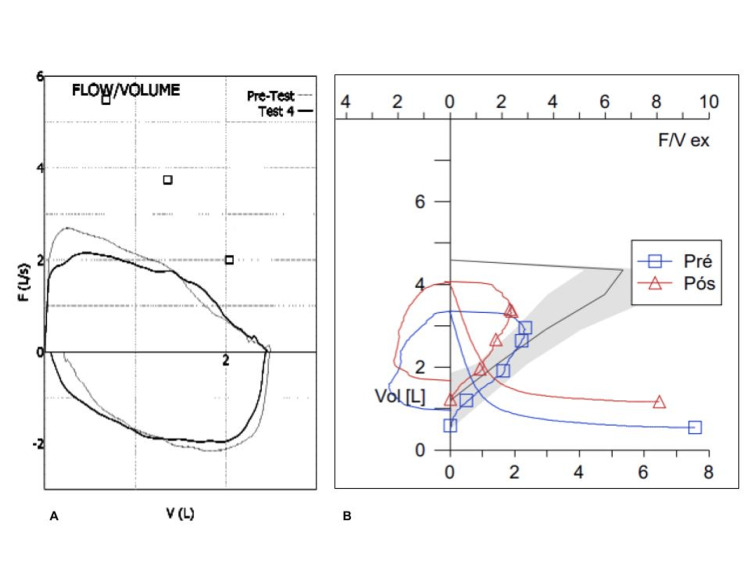
Flow-volume curves from the respiratory function tests performed at 13 (A) and 19 (B) years of age. Fixed airflow obstruction is evident from the flattening of the inspiratory and expiratory flow-volume curves, with a slight worsening over time.

**Figure 2:**
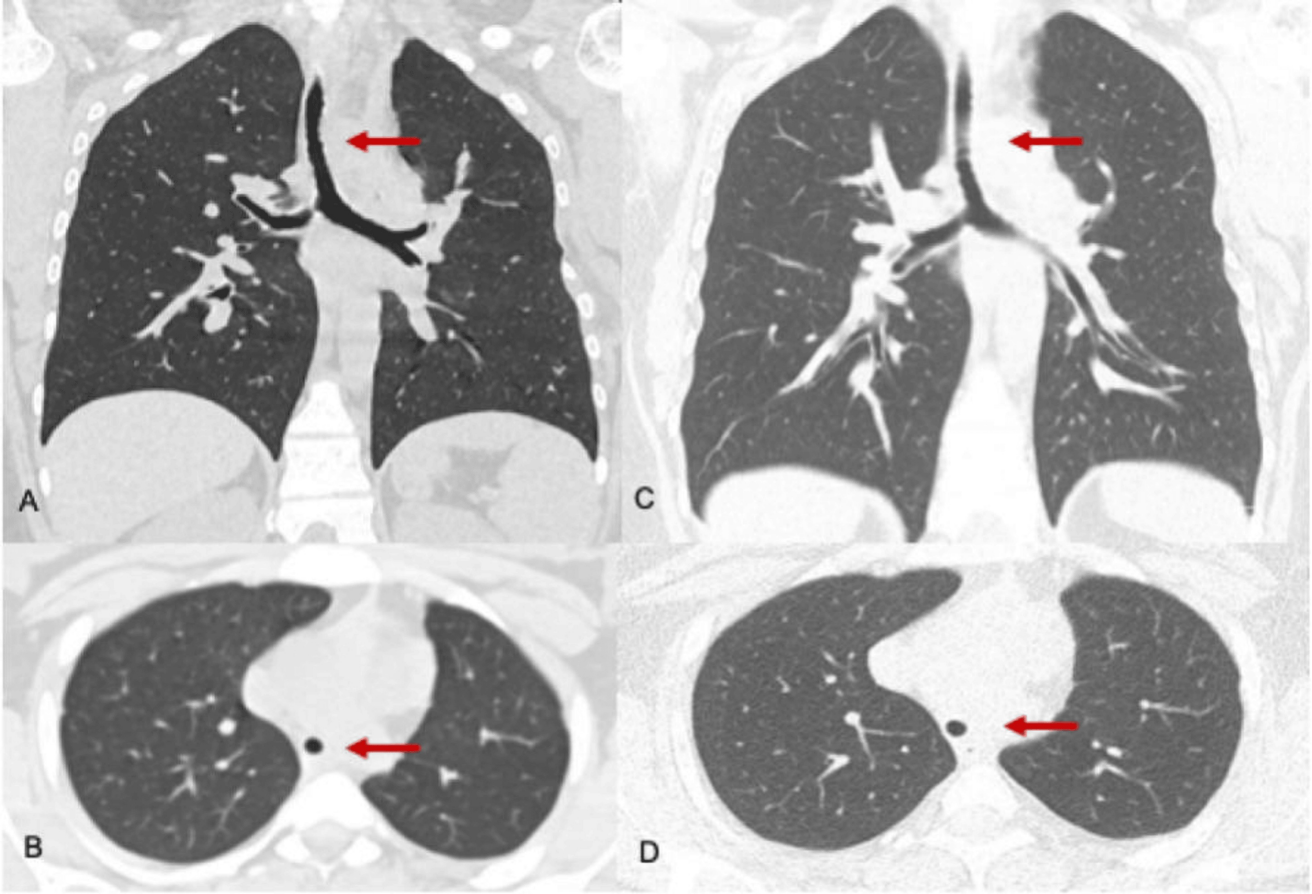
Coronal and sagittal CT scans of the chest demonstrating stable diffuse tracheal obstruction at 13 (A, B) and 19 (C, D) years of age. The images show persistent diffuse narrowing of the thoracic trachea, with a minimum luminal diameter of 6-7 mm.

During childhood, she had no recurrent respiratory infections but developed episodes of shortness of breath and stridor with moderate exertion, which gradually became more frequent. A trial of inhaled corticosteroids led to partial improvement. At 13 years of age, pulmonary function tests (Table 1) showed an obstructive pattern without a bronchodilator response (forced vital capacity (FVC) 2500 mL, 92% predicted; forced expiratory volume in one second (FEV₁) 1890 mL, 70%; FEV₁/FVC ratio 76%; maximal mid-expiratory flow between 25% and 75% of FVC (MMEF₂₅-₇₅) 1650 mL, 51%; post-bronchodilator: FVC 2460 mL, 90%; FEV₁ 1840 mL, 68%; FEV₁/FVC 75%; MMEF₂₅-₇₅ 1710 mL, 53%), with flattening of both inspiratory and expiratory flow-volume curves (Figure 1A). Chest computed tomography (CT) revealed a circular tracheal shape with posterior indentation, consistent with congenital tracheal stenosis. The average tracheal diameter was 6-8 mm, and the segmental bronchi appeared slightly smaller in caliber compared with their accompanying vessels (Figure 2A-2B). The diagnosis was confirmed by flexible bronchoscopy, which showed complete tracheal rings and diffuse narrowing of the trachea.

**Table 1:** Pulmonary function tests (PFTs) of the patient at 13 and 19 years of age. Values are presented as absolute volumes (mL) and percent predicted. Predicted (normal) values are approximate, based on the GLI 2012 reference equations for age, sex, and height. Values below 80% of predicted indicate airway obstruction. No significant bronchodilator response was observed, consistent with fixed tracheal narrowing. Over time, the overall obstructive pattern remained, with a slight reduction in airflow. Pre-BD: pre-bronchodilator, post-BD: post-bronchodilator.

Parameter	Approximated predicted (normal)	13 years pre-BD	19 years post-BD	13 years pre-BD	19 years post-BD
FVC (mL/%)	2460	2500/92	2460/90	2810/82	2900/85
FEV1 (mL/%)	2100	1890/70	1840/68	2050/68	1970/65
FEV1/FVC (%)	86	76	75	73.72	67.92
MMEF₂₅–₇₅ (mL/%)	1900	1650/51	1710/53	1580/43	1680/46

Given her stable clinical course, she remained under surveillance and was transitioned to adult pulmonology at 19 years of age. At that time, she reported daily stridor and exertional dyspnea, with partial relief from inhaled bronchodilators. Pulmonary function testing (Table 1) demonstrated progression of obstruction (FVC 2810 mL, 82% predicted; FEV₁ 2050 mL, 68%; FEV₁/FVC 73%; MMEF₂₅-₇₅ 1580 mL, 43%; post-bronchodilator: FVC 2900 mL, 85%; FEV₁ 1970 mL, 65%; FEV₁/FVC 68%; MMEF₂₅-₇₅ 1680 mL, 46%) and persistent flattening of the flow-volume loops (Figure 1B). Follow-up CT confirmed diffuse narrowing of the thoracic trachea, with a minimum diameter of 6-7 mm, and similarly reduced caliber of the intrapulmonary bronchi, without other structural abnormalities (Figure 2C-2D).

**Figure 3:**
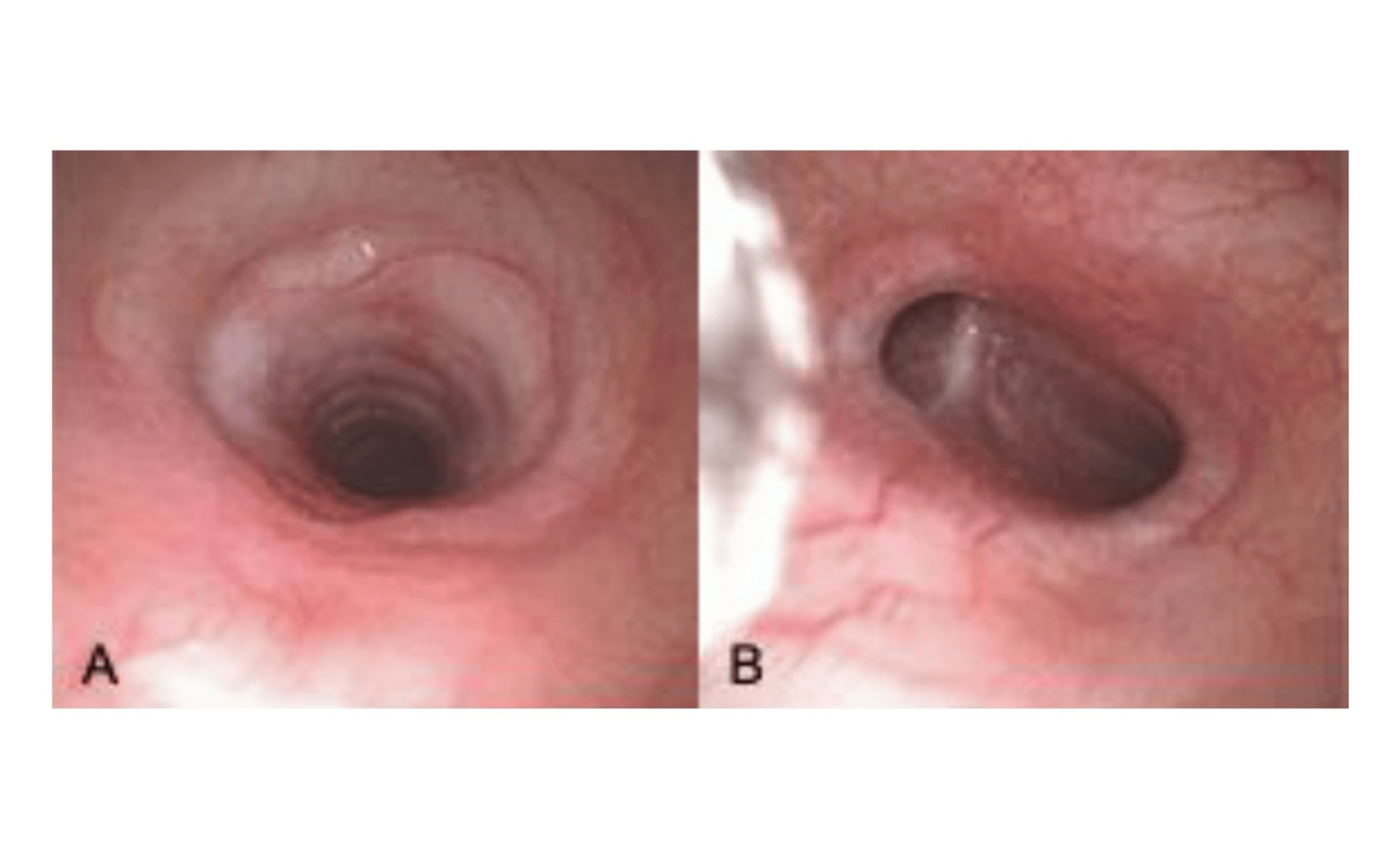
Bronchoscopy image showing diffuse tracheal obstruction: (A) upper third of the trachea, (B) at the level of the carina.

On re-evaluation, flexible bronchoscopy demonstrated complete cartilaginous rings causing diffuse stenosis along the entire trachea, with no focal areas suitable for endoscopic intervention (Figure 3A-3B).

A six-minute walk test showed a distance of 425 m (70% predicted) without significant desaturation, with peripheral oxygen saturation of 96% at baseline and 93% at the end of the test. After multidisciplinary evaluation with the thoracic surgery team, conservative management with regular follow-up was recommended, considering her clinical stability and the surgical risk.

## Discussion

We report a case of CTS associated with pulmonary artery sling in an adult. Pulmonary artery sling is frequently accompanied by CTS due to their direct anatomical and embryological relationship [7,8]. This association largely determines the severity of respiratory symptoms and often requires a combined surgical approach to restore airway patency [1,9]. However, when tracheal stenosis is mild (tracheal diameter > 3 mm), and symptoms are minimal or absent, isolated reimplantation of the pulmonary artery may be sufficient, as may be the case in this patient [10,11].

Diagnosis and monitoring rely on clinical assessment, imaging, and endoscopic confirmation. Early respiratory symptoms, such as stridor, dyspnea, choking, or exercise limitation, should raise suspicion, particularly in childhood. CT provides a detailed assessment of the tracheal anatomy, stenosis extent, circular morphology, and associated bronchial anomalies [8,12]. Bronchoscopy remains the gold standard for confirming complete tracheal rings, defining stenosis severity, differentiating congenital from acquired lesions, and planning treatment [12].

Pulmonary function testing aids differential diagnosis and monitoring. Symptoms such as wheezing and exercise limitation can mimic asthma. In this patient, flow-volume loops demonstrated classic flattening of inspiratory and expiratory limbs, consistent with fixed tracheal obstruction, with reduced peak inspiratory and expiratory flows (PIF and PEF) [13]. Reduced FEV₁, FVC, and MMEF₂₅-₇₅ without bronchodilator response indicated persistent obstruction, potentially marking progression of central airway stenosis [1].

Given the potential for clinical deterioration suggested by pulmonary function tests and exertional limitation, follow-up CT and bronchoscopy were performed. Both examinations showed stable findings, supporting further discussion in a multidisciplinary team meeting. Surgical intervention, such as slide tracheoplasty, depends on symptom severity, stenosis extent, and risk of respiratory compromise. In this case, diffuse tracheal stenosis with a minimal diameter of 6-7 mm, no focal lesions suitable for endoscopic treatment, and relative clinical stability, a conservative management with regular follow-up was considered appropriate. Exercise tolerance was preserved, with no significant oxygen desaturation. Tracheoplasty can be technically challenging in diffuse, extensive stenoses [2,14,15]. Inhaled corticosteroids may be considered as adjunctive therapy for symptom control, though evidence is limited to small case series [16].

## Conclusions

This case of CTS associated with pulmonary artery sling highlights the diagnostic and therapeutic challenges of this rare condition and underscores the need for a multidisciplinary, individualized approach. Regular clinical and functional follow-up is essential to monitor complications and optimize outcomes. Early diagnosis, the use of advanced imaging and endoscopic techniques, and long-term surveillance are critical for achieving the best results in patients with rare and potentially life-threatening malformations. There is still no consensus on optimal follow-up intervals or long-term outcomes in adults with persistent diffuse congenital tracheal stenosis, emphasizing the need for further studies on its natural history, functional impact, and quality of life.
